# Persistence of gut dysbiosis in individuals with anorexia nervosa

**DOI:** 10.1371/journal.pone.0296037

**Published:** 2023-12-20

**Authors:** Yukiko Morisaki, Noriyuki Miyata, Megumi Nakashima, Tomokazu Hata, Shu Takakura, Kazufumi Yoshihara, Takafumi Suematsu, Koji Nomoto, Kouji Miyazaki, Hirokazu Tsuji, Nobuyuki Sudo

**Affiliations:** 1 Department of Psychosomatic Medicine, Graduate School of Medical Sciences, Kyushu University, Fukuoka, Japan; 2 Center for Health Sciences and Counseling, Kyushu University, Fukuoka, Japan; 3 Faculty of Life Sciences, Department of Molecular Microbiology, Tokyo University of Agriculture, Setagaya City, Japan; 4 Yakult Central Institute, Tokyo, Japan; Universidad San Francisco de Quito, ECUADOR

## Abstract

Recent evidence suggests a crucial role of the gut microbiota in the pathogenesis of anorexia nervosa (AN). In this study, we carried out a series of multiple analyses of the gut microbiota of hospitalized individuals with AN over three months using 16S or 23S rRNA-targeted reverse transcription–quantitative polymerase chain reaction (PCR) technology (YIF-SCAN^®^), which is highly sensitive and enables the precise quantification of viable microorganisms. Despite the weight gain and improvements in psychological features observed during treatment, individuals with AN exhibited persistent gut microbial dysbiosis over the three-month duration. Principal component analysis further underscored the distinct microbial profile of individuals with AN, compared with that of age-matched healthy women at all time points. Regarding the kinetics of bacterial detection, the detection rate of *Lactiplantibacillus* spp. significantly increased after inpatient treatment. Additionally, the elevation in the *Bifidobacterium* counts during inpatient treatment was significantly correlated with the subsequent body weight gain after one year. Collectively, these findings suggest that gut dysbiosis in individuals with AN may not be easily restored solely through weight gain, highlighting the potential of therapeutic interventions targeting microbiota via dietary modifications or live biotherapeutics.

## Introduction

Anorexia nervosa (AN) is characterized by severe weight loss and a pathological fear of weight gain [1, 2]. It affects a notable percentage of women, with a prevalence ranging from 1% to 4% [3]. AN is a psychiatric disorder with one of the highest mortality rates among mental illnesses [4]. Clinically, AN is classified into restricting-type AN (ANR) and binge/purge-type AN (ANBP). Individuals with ANR engage in extreme food restrictions, leading to pronounced physical and psychiatric symptoms resulting from severe emaciation. Individuals with ANBP frequently exhibit episodes of compulsive overeating or engage in purging behaviors, such as self-induced vomiting, in addition to severe dietary restriction.

Psychosocial factors have long been acknowledged as significant contributors to the etiology and progression of eating disorders [5–7]. However, biological factors substantially influence the pathology of AN [8, 9]. A recent large-scale genome-wide association study [10] has reported that genetic underpinnings of AN encompass both metabolic and psychiatric components, challenging the previously held notion of AN having a purely psychiatric origin. Consequently, genetically predisposed individuals have been postulated to be susceptible to the development of AN when exposed to specific environmental factors, including psychosocial ones. In this context, the gut microbiota has emerged as a potential environmental factor that can profoundly affect the pathological processes associated with AN [11–13]. Multiple independent research groups, including ours, have reported gut dysbiosis in individuals with AN [13–18]. For example, in our study of gut microbes, compared with age-matched healthy women, women with AN harbored fewer total bacteria and obligate anaerobes, including those from the *Blautia (Clostridium) coccoides* group, *Clostridium leptum* subgroup, and *Bacteroides fragilis* group [18]. Moreover, the serum metabolic profiles of individuals with AN differed from those of healthy women, exhibiting decreased amino acid levels and elevated concentrations of uremic toxins [19]. Notably, such dysbiosis contributed to poor weight gain and anxiety-like behavior in an animal model of AN wherein the gut microbiota from individuals with AN was transplanted [20]. Hence, the gut microbiota is a critical determinant influencing the development and clinical trajectory of AN. However, a crucial gap in the current understanding pertains to how the aberrant gut microbiota observed in individuals with AN re-establishes with weight gain, as this knowledge bears significant therapeutic implications. If a patient’s gut microbiota remains unchanged despite weight restoration, targeting it through dietary modifications or live biotherapeutic interventions [21] could be a therapeutic option.

In this study, we conducted a series of multiple analyses of the gut microbiota in hospitalized individuals over three months to analyze fluctuations in the gut microbiota in relation to weight gain among individuals with AN. Additionally, we explored the correlation between these alterations in the gut microbiota and the maintenance of body weight at one year after treatment commencement.

## Materials & methods

### Subjects

We enrolled Japanese individuals with AN who were admitted to or visited our outpatient department at Kyushu University Hospital between May 2, 2011, and August 1, 2013; 14 and 11 women with ANR and ANBP, respectively, agreed to participate in this study. Among these 25 individuals, 7 and 6 patients with ANR and ANBP, respectively, aged 27.7 ± 10.8 years were subsequently followed up by the current study after obtaining their consent. Mean duration of the disease was 8.8 ± 8.4 years (0.3–27.3 years). Among the 13 participants, a variety of prior therapeutic interventions were noted, including cases without treatment as well as those with inpatient nutritional therapy, cognitive behavioral treatment, supportive psychotherapy, or a combination of these therapeutic modalities. We excluded participants with the following conditions: severe physical diseases (such as renal failure and infectious diseases), a history of antibiotic use, or regular consumption of yogurt or probiotics within three months before the study began. Individuals with AN underwent structured interviews, and their current AN phenotypes were diagnosed according to the Diagnostic and Statistical Manual of Mental Disorders-IV-TR criteria. To compare individuals with AN to healthy women, we used the same subjects previously reported as healthy controls (CON) [18].

The study protocol was approved by the Institutional Ethics Review Board of Kyushu University Hospital (Permission No. 23–7; validity period, May 2, 2011 to March 31, 2014), and written informed consent was obtained from all participants before enrollment. If participants were minors, consent was obtained from their parents.

### Self-reported questionnaires

All participants completed a battery of self-reported questionnaires three or four times over a three-month period. Depression and anxiety levels were evaluated using the Japanese versions of the Center for Epidemiologic Studies-Depression Scale (CESD) [22] and State-Trait Anxiety Inventory (STAI) [23], respectively. Psychopathology related to eating disorders was assessed using the Japanese version of the Eating Disorder Inventory (EDI) [24].

### Bacterial enumeration through Yakult Intestinal Flora-SCAN (YIF-SCAN^®^)

Fecal samples were collected from the participants at various time points, including 0 (baseline, n = 13), 1 (n = 13), 2 (n = 12), and 3 (n = 8) months after baseline. The baseline was determined to be approximately one or two weeks after the initial admission day, as frequent day-to-day fluctuations occurred in body weight immediately after admission. A total of 13 individuals with AN consented to participate in the follow-up study. Within this cohort, all 13 participants submitted fecal samples one month after the commencement of the treatment protocol. Subsequently, 1 participant opted to discontinue providing fecal specimens two months into the treatment, while an additional 4 patients declined to provide fecal samples altogether. Consequently, only 8 out of the 13 individuals completed fecal sampling protocol. Fecal samples were processed according to a previously described methodology [25]. Briefly, total RNA fractions were extracted from fecal samples by a previously established protocol. The composition of the major gut bacterial groups was assessed using the YIF-SCAN^®^ technique, which employs 16S or 23S rRNA-targeted reverse transcription quantitative PCR (RT-qPCR) technology [18, 26–29].

### Quantification of organic acids and pH levels in fecal samples

Fecal organic acids were quantified using previously established methods [30]. In brief, the fecal samples were homogenized in 0.15 mol/L perchloric acid, and the resulting suspension was collected after centrifugation at 20,400 × g for 10 min at 4°C. The concentrations of organic acids in the samples were measured using a high-performance liquid chromatography system (432 Conductivity Detector; Waters Co., Milford, MA, USA). Additionally, fecal pH levels were determined using an IQ 150 pH/thermometer (IQ Scientific Instruments, Inc., Carlsbad, CA).

### Statistical analysis

Continuous data are presented as means ± standard deviations (SDs). Statistical analyses were performed using the JMP PRO v.17 software package for Windows (SAS Institute, Japan).

To evaluate the effect of time on changes in each bacterial count and short chain fatty acid (SCFA) level or alterations in psychological parameters, we used a repeated-measures analysis of variance (ANOVA) followed by the Bonferroni correction based on the number of tests. The detection rate of *Lactiplantibacillus* spp. (formerly *Lactobacillus plantarum* subgroup) was calculated as the ratio of individuals harboring the bacterium to the total number of individuals in the group, and the change in *Lactiplantibacillus* spp. detection rate was evaluated using Fisher’s exact test. Pearson correlation coefficients were used to assess the correlation between increased *Bifidobacterium* counts and an increase in body weight at one year after the commencement of inpatient treatment. The elevated *Bifidobacteria* counts were determined by calculating the difference between the baseline and three-month values. However, in the case of four individuals with AN, data for three-month assessments were unavailable. Therefore, the correlation analysis for these cases was instead conducted using the difference between the baseline and two-month values.

Principal component analysis (PCoA) was performed using the log-transformed bacterial counts. For cases in which bacteria were not detected, the corresponding primer sets’ detection limits were considered half of the bacterial counts (**S1 Table**). The following counts were included in the analysis: total bacteria, *Blautia coccoides* group, *C*. *leptum* subgroup, *Bacteroides fragilis* group, *Bifidobacterium*, *Atopobium* cluster, *Prevotella*, *Enterobacteriaceae*, *Enterococcus*, *Staphylococcus*, *Streptococcus*, *Clostridioides (Clostridium) difficile*, *Clostridium perfringens*, and total lactobacilli. As the first principal component values exhibited a normal distribution, confirmed by the Shapiro–Wilk normality test, we compared the differences between the CON and AN groups at different time points using unpaired Student’s t-tests based on the first principal component values. Permutational multivariate ANOVA (PERMANOVA) [31, 32] was also conducted to evaluate differences in bacterial composition between the CON and AN groups. This was performed using the Adonis function of the vegan package in R studio with R 3.6.2. Comparisons between two groups (CON vs. ANR, CON vs. ANBP, or ANR vs. ANBP) were performed using the Wilcoxon-Mann-Whitney test followed by Bonferroni correction based on the number of tests.

## Results

### Time-course changes in body weight in individuals with AN

**Table 1** presents the dynamic fluctuations in body weight and BMI in a cohort of 13 individuals with AN. Repeated-measures ANOVA revealed consistent increases in body weight (F_(5, 57)_   = 14.1, p < 0.0001) and BMI (F_(5, 57)_   = 13.9, p < 0.0001). Specifically, the average weight of individuals increased from 29.7 kg at the commencement of inpatient treatment to 40.2 kg one year after the initiation of the study. Nevertheless, 3 out of the 13 individuals displayed minimal or limited improvement in body weight during this time frame, as evidenced by their BMI values that remained below 15.

**Table 1:** Time-course changes in body weight and BMI in individuals with AN†.

ID No.		Basal	1 month	2 months	3 months	12 months
A	BW	27.15	29.0	31.0	33.9	38.8
	BMI	11.2	11.9	12.7	13.9	15.9
B	BW	30.1	29.9	31.6	34.0	34.5
	BMI	11.6	11.5	12.2	13.1	13.3
C	BW	33.1	35.3	40.0	41.2	33.3
	BMI	12.3	13.1	14.9	15.3	12.4
D	BW	36.8	38.3	41.0	44.0	47.8
	BMI	14.2	14.8	15.9	17.0	18.4
E	BW	29.4	32.4	35.0	33.4	35.9
	BMI	12.6	13.9	15.0	14.3	15.3
F	BW	30.8	31.5	34.1	36.1	41.3
	BMI	12.3	12.6	13.6	14.4	16.5
G	BW	35.9	36.3	38.0	39.7	43.0
	BMI	13.2	13.3	14.0	14.6	15.8
H	BW	29.6	33.7	35.1	35.7	37.6
	BMI	12.2	13.8	14.4	14.7	15.5
I	BW	22.5	24.9	27.0	28.3	27.9
	BMI	10.3	11.4	12.3	12.9	12.7
J	BW	26.9	28.3	31.0	35.9	38.9
	BMI	12.4	13.1	14.3	16.6	18.0
K	BW	31.1	29.3	31.2	32.4	41.3
	BMI	12.3	11.6	12.4	13.2	16.4
L	BW	25.9	28.0	31.5	32.4	45.1
	BMI	10.5	11.3	12.8	13.1	18.3
M	BW	27.3	29.3	31.7	33.0	57.3
	BMI	12.0	12.8	13.9	14.5	25.1
Mean	BW	29.7 (4.0)	31.2 (3.8)	33.7 (4.0)	35.4 (4.2)	40.2 (7.3)
(SD)	BMI	12.1 (1.0)	12.7 (1.1)	13.7 (1.2)	14.4 (1.2)	16.4 (3.3)

**†**Body weight (BW, kg) and body-mass index (BMI, kg/m^2^) at baseline, 1, 2, 3, and 12 months after the start of treatment are shown.

### Kinetics of psychological parameters

Scores of the psychological tests, including CESD, EDI, and EDI subscale “bulimia,” exhibited statistically significant improvements during the three-month observation period, compared with the baseline scores (**Table 2**).

**Table 2:** Kinetics of psychological parameters†.

	Basal	1 month	2 months	3 months	p value
	(n = 13)	(n = 12)	(n = 12)	(n = 9)	
CESD	25.9 (11.3)	16.8 (12.9)	**15.6 (10.0)** *	14.8 (13.6)	**0.0021**
STAI state	48.0 (11.5)	45.4 (14.8)	41.9 (13.5)	42.6 (15.7)	0.1729
STAI trait	56.9 (14.3)	51.3 (15.4)	50.6 (15.3)	42.6 (15.2)	0.0190
EDI	77.4 (29.4)	53.3 (27.0)	52.2 (29.2)	53.6 (29.6)	**0.0004**
Drive for thinness	9.8 (7.3)	5.2 (6.9)	5.3 (6.6)	6.6 (7.4)	0.0095
Interoceptive awareness	10.3 (7.3)	7.0 (7.3)	4.5 (5.8)	5.9 (6.3)	0.0299
Bulimia	5.9 (4.6)	1.9 (3.2)	**0.9 (2.3)** **	1.8 (3.4)	**0.0004**
Body dissatisfaction	13.6 (5.4)	13.9 (4.9)	13.5 (7.8)	15.2 (7.0)	0.9825
Ineffectiveness	15.1 (8.8)	11.3 (6.9)	11.0 (7.1)	9.9 (7.2)	0.0360
Maturity fears	8.6 (6.1)	6.1 (4.8)	6.9 (5.1)	6.2 (5.9)	0.0569
Perfectionism	4.5 (4.3)	3.0 (4.0)	4.0 (4.8)	3.7 (4.5)	0.2093

**†**All data are expressed as means (SDs). A repeated-measures ANOVA was conducted to examine the effects of time on the psychological parameter values. The results were corrected using the Bonferroni test, based on the number of trials. For comparisons between the baseline and specific variables, the Steel test was employed when the p value obtained from the ANOVA was <0.0041 (0.05/12).

** p *< 0*.*01* and

* p *< 0*.*05* indicate a significant difference between the basal value and the indicated variable.

### Time-course changes in the composition of gut microbes and SCFA levels

Repeated-measures ANOVA with Bonferroni correction identified no statistically significant alterations observed in any of the bacterial species over the course of three months following the initiation of treatment (**Table 3**). Similarly, the concentrations of SCFA and pH levels in the fecal samples showed no significant changes throughout the specified duration (**Table 4**). Regarding AN phenotypes, individuals with ANR at baseline did not show any difference in gut microbes when compared with individuals with ANBP at baseline (**S2 Table**).

**Table 3:** Kinetics of the number of gut microbiota†.

	log_10_ cells/g feces				
	Basal	1 month	2 months	3 months	p value
	(n = 13)	(n = 13)	(n = 12)	(n = 8)	
Total bacterial count	10.6 (0.4)	10.7 (0.4)	10.8 (0.3)	10.5 (0.5)	0.6825
*Blautia coccoides* group	9.4 (0.4)	9.3 (0.5)	9.3 (0.6)	9.1 (0.5)	0.9597
*C*. *leptum* subgroup	9.6 (0.4)	9.7 (0.5)	9.6 (0.6)	9.6 (0.7)	0.9166
*B*. *fragilis* group	9.6 (0.5)	9.3 (0.8)	9.6 (0.6)	9.3 (0.5)	0.5469
*Bifidobacterium*	10.1 (1.2)	10.4 (0.6)	10.5 (0.6)	10.2 (0.7)	0.4528
*Atopobium cluster*	9.1 (1.3)	9.3 (1.0)	9.1 (1.2)	9.8 (0.4)	0.5789
*Prevotella*	6.5 (1.1)	6.3 (1.0)	6.1 (1.0)	7.4 (2.0)	0.2151
*Enterobacteriaceae*	7.2 (0.9)	6.4 (1.0)	6.9 (0.9)	6.5 (0.8)	0.0401
*Enterococcus*	6.9 (1.1)	7.2 (1.5)	6.8 (1.7)	7.1 (1.5)	0.4857
*Staphylococcus*	5.8 (0.8)	5.6 (1.0)	5.6 (0.6)	5.8 (0.7)	0.8403
*Streptococcus*	8.4 (0.7)	8.2 (0.9)	8.2 (0.8)	8.4 (0.8)	0.2517
*C*. *perfringens*	5.0 (1.5)	5.0 (1.0)	5.3 (1.8)	5.4 (2.7)	0.3733
*Clostridioides difficile*	5.8 (0.7)	5.0 (0.6)	ND	ND	NT
Total lactobacilli	6.3 (2.3)	6.7 (1.9)	6.5 (1.6)	6.0 (1.1)	0.7640
*Lactobacillus*	5.1 (1.9)	5.2 (1.7)	6.2 (1.2)	4.9 (1.4)	0.7523
*Lactiplantibacillus*	4.0 (1.6)	5.4 (2.2)	4.0 (0.8)	3.6 (0.4)	0.8702
*Limosilactobacillus except L*. *fermentum*	5.0 (1.7)	4.9 (1.2)	5.2 (1.4)	4.3 (0.8)	0.8589
*Lacticaseibacillus*	6.8 (1.9)	7.0 (1.7)	6.2 (1.3)	6.0 (1.6)	0.4279
*Liquorilactobacillus and Ligilactobacillus*	5.6 (1.6)	5.1 (2.2)	4.4 (1.5)	3.7 (0.5)	0.6983
*Latilactobacillus*	3.7 (0.5)	4.2 (0.8)	3.8 (1.0)	3.9	0.9461
*Limosilactobacillus*	8.1 (0.8)	7.3 (1.4)	6.8 (1.1)	5.7 (0.9)	0.9854
*Levilactobacillus*	3.9	3.4 (0.8)	4.5	4.2	NT

**†**All data are expressed as means (SDs). ND, not detected; NT, not tested; *C*, *Clostridium; B*, *Bacteroides*. Total lactobacilli is expressed as the sum of the counts of *Lactobacillus*, *Lactiplantibacillus*, *Limosilactobacillus except L*. *fermentum*, *Lacticaseibacillus*, *Liquorilactobacillus and Ligilactobacillus*, *Latilactobacillus*, *Limosilactobacillus*, and *Levilactobacillus*. Repeated-measures ANOVA was conducted to examine the effects of time on each bacterial count. The results were corrected using the Bonferroni test, based on the number of trials; therefore, p values of <0.0025 (0.05/20) were considered significant.

**Table 4:** Kinetics of fecal SCFA levels†.

	μmol/g feces				
	Basal	1 month	2 month	3 month	p value
	(n = 13)	(n = 12)	(n = 12)	(n = 9)	
Total organic acids	50.7 (21.4)	39.5 (17.8)	56.5 (26.0)	60.2 (34.0)	0.1998
Succinic acid	7.0 (13.7)	0.5 (0.6)	0.8 (1.7)	0.7 (1.30)	0.0562
Lactic acid	0 (0)	0 (0)	1.6 (4.9)	0.2 (0.7)	0.3411
Formic acid	0.6 (1.2)	0.6 (0.9)	0.9 (1.1)	0.7 (1.1)	0.8139
Acetic acid	28.8 (12.1)	25.3 (12.5)	34.8 (13.8)	38.8 (21.6)	0.0731
Propionic acid	8.9 (5.2)	7.4 (4.2)	9.1 (5.8)	10.2 (7.7)	0.6802
Butyric acid	1.4 (2.1)	1.9 (2.7)	4.0 (5.9)	2.5 (3.5)	0.3668
Iso-valeric acid	0.9 (1.9)	1.8 (2.5)	1.4 (2.3)	1.6 (2.5)	0.6290
Valeric acid	0.5 (1.7)	0.5 (1.4)	0.8 (1.8)	1.0 (2.0)	0.6648
pH	7.3 (0.9)	7.7 (0.6)	7.4 (0.6)	7.5 (0.6)	0.1651

**†**All data are expressed as means (SDs). Repeated-measures ANOVA was conducted to examine the effects of time on each SCFA count.

### Comparative analysis of gut bacteria in healthy women and individuals with AN

**Fig 1:**
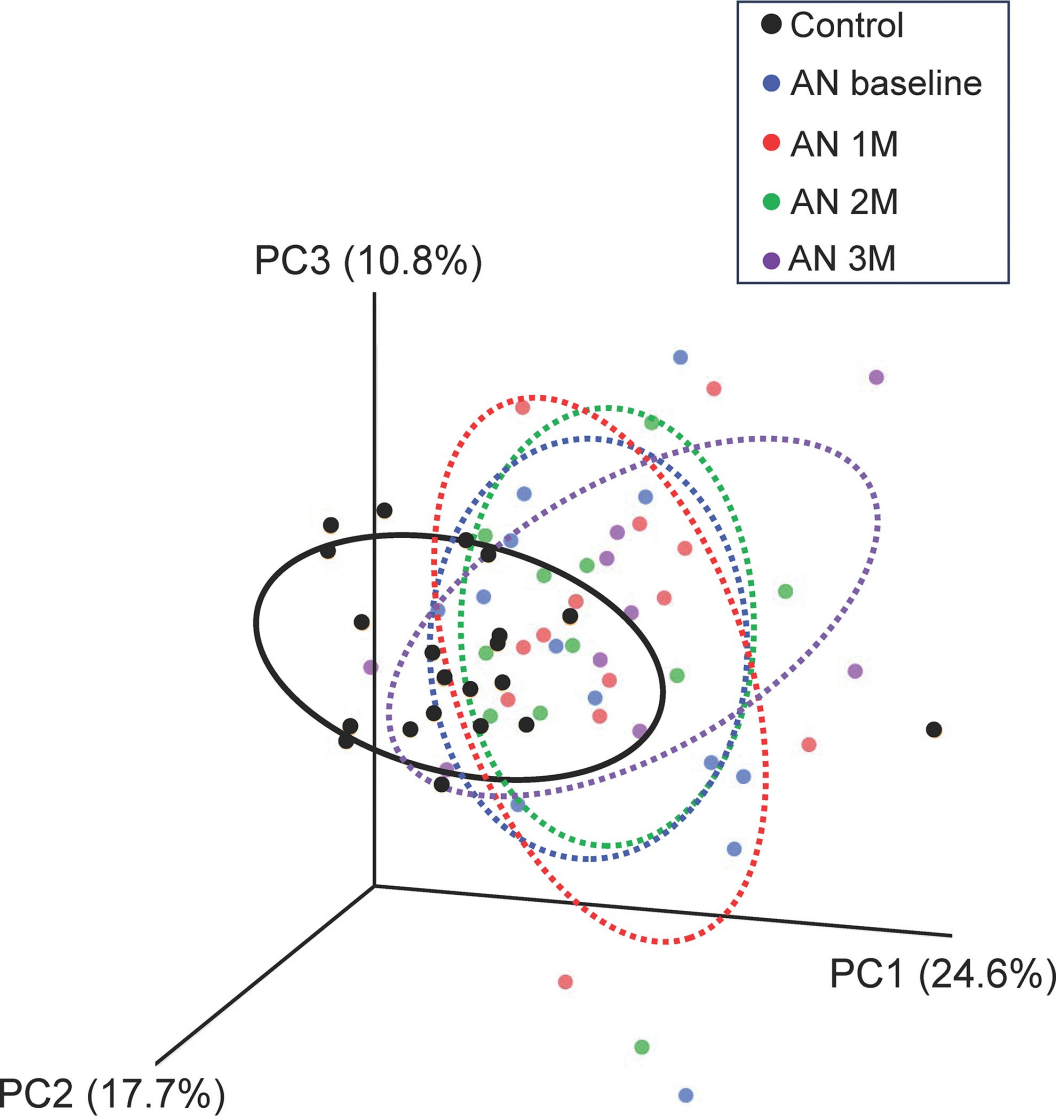
Principal component analysis of bacterial counts in healthy female controls and individuals with anorexia nervosa (AN). Black, blue, red, green, and purple plots show the data for controls, patients with AN at baseline and 1 month (1M), 2 months (2M), and 3 months (3M) after the start of treatment, respectively. Each colored ellipse represents 50% of the samples belonging to a cluster. Explained variances are shown in parentheses.

Our prior investigation [18] revealed marked distinctions in the gut microbial composition between individuals with AN and the age-matched CON group. These findings were reverified by a comparison involving the CON group and 13 individuals with AN at the baseline of this study (**S3 Table**). **Fig 1** shows a three-dimensional PCoA that effectively illustrates the dissimilarities between the AN group cluster and the CON group. This observation was further confirmed by the application of unpaired Student’s t-tests to the first principal component values. The CON group differed significantly from the AN group across all time points [CON vs. AN baseline, t(62)   = -3.7, p = 0.0005; CON vs. AN 1 month, t(62)   = -3.9, p = 0.0003; CON vs. AN 2 months, t(62)   = -3.2, p = 0.0023; CON vs. AN 3 months, t(62)   = -3.8, p = 0.0003]. In contrast, no significant differences were observed between any two groups selected from the four AN groups at different time points. These results were also confirmed by PERMANOVAs that showed a significant difference between the CON group and the AN group across all time points [CON vs. AN baseline, f = 19.9, p = 0.001; CON vs. AN 1 month, f = 21.2, p = 0.001; CON vs. AN 2 months, f = 18.2, p = 0.001; CON vs. AN 3 months, f = 14.4, p = 0.001].

### Detection rate of *Lactiplantibacillus* spp. increased with weight gain

In our previous study, we observed a noteworthy disparity in the detection rates of *Lactiplantibacillus* spp. between individuals with AN and the CON group [18]. Therefore, we investigated whether an increase in body weight led to an increase in the detection rate of *Lactiplantibacillus* spp.

Consequently, the detection rate of *Lactiplantibacillus* spp. was significantly augmented during the three-month inpatient treatment period (detection rate: baseline, 25%; 1 month, 15.4%; 2 months, 66.7%; 3 months, 44.4%) when analyzed using Fisher’s exact test (two-tailed p = 0.0485). However, no other bacteria exhibited significant changes in detection rates during this period.

### Increased number of *Bifidobacterium* correlates with weight gain during the one-year period

**Fig 2:**
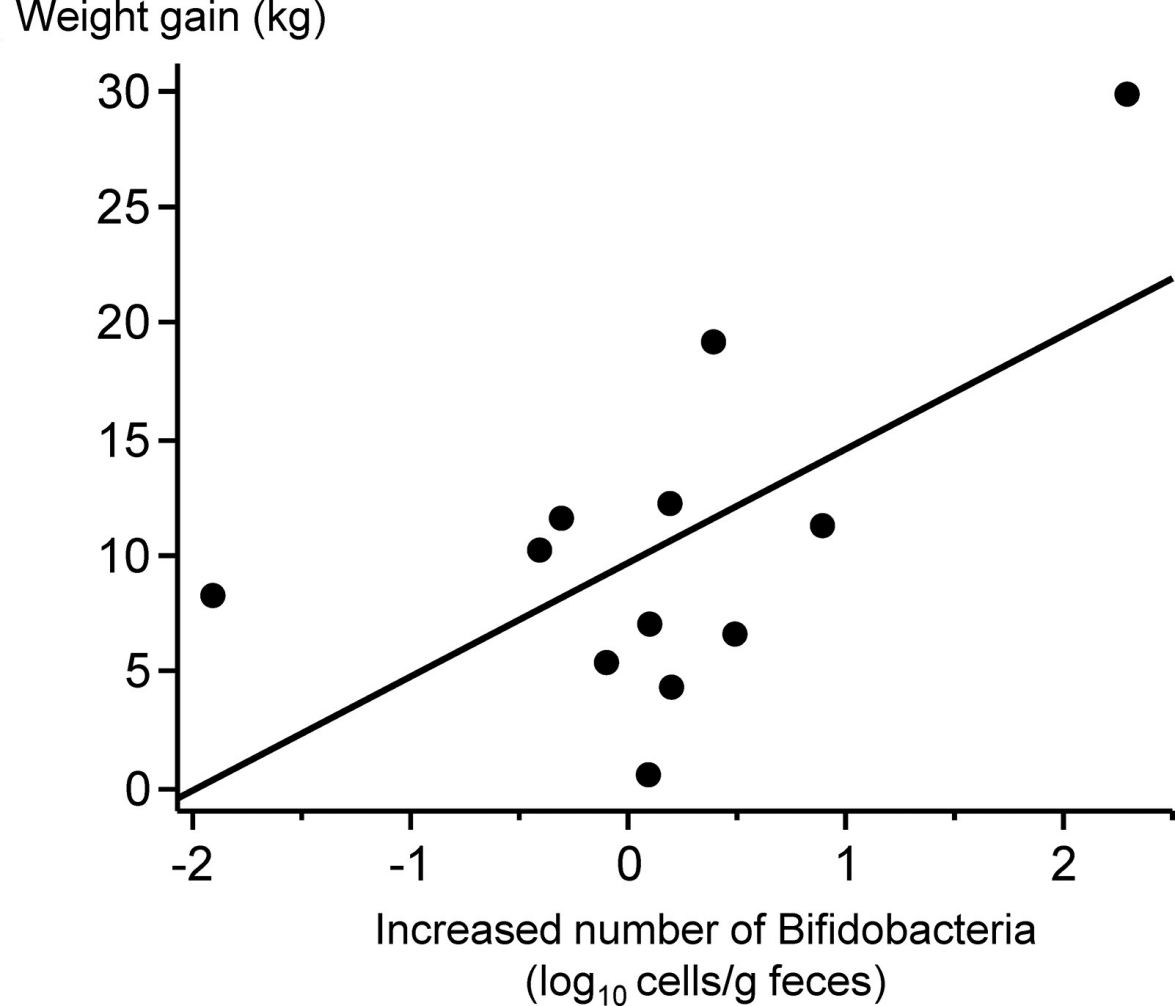
Increased number of *Bifidobacterium* during inpatient treatment correlates with weight gain one year after treatment. The relationship between the increased number of *Bifidobacterium* during the first 2 or 3 months of treatment and the increase in body weight one year after the start of inpatient treatment was analyzed using Pearson correlation.

The increase in *Bifidobacterium* counts during the first two or three months following the beginning of treatment was not associated with weight gain during the same period. Nevertheless, a significant correlation was identified between the increase in *Bifidobacterium* populations and the increase in body weight one year after the start of inpatient treatment (**Fig 2**, Pearson’s correlation coefficient, r = 0.605, p = 0.0371). No additional factors, including other bacterial or psychological parameters, were found to influence the increase in body weight at the end of the 1-year period.

## Discussion

In this study, individuals with AN showed persistent gut dysbiosis during inpatient treatment despite increased body weight and improved psychological features. The PCoA also exhibited a distinct profile in individuals with AN, compared with that in age-matched healthy women. The detection rate of *Lactiplantibacillus* spp. significantly increased during the study period. Notably, a significant correlation was found between the increase in the *Bifidobacterium* count and the increase in body weight after one year. These findings suggest that the dysbiosis found in individuals with AN may not be solely restored by weight gain or caloric intake and highlight the need for comprehensive treatment approaches targeting both weight restoration and gut microbiota modulation.

Our current results, in which the gut microbiota remained unchanged even after weight restoration in individuals with AN, are consistent with previous studies using the 16S rRNA gene for taxonomic differentiation [13, 16, 17, 33]. Moreover, a recent study using shotgun metagenomic sequencing [34] also revealed notable dissimilarities between the gut microbiota of individuals with AN and those of individuals without eating disorders, corroborating the present results using 16S or 23S rRNA-targeted RT-qPCR technology. Taken together, these findings provide a rationale for microbiota-targeted therapeutic interventions, such as probiotics, for treating AN that is refractory to ordinary treatment options.

Our previous study [20] investigated whether gut dysbiosis in individuals with AN could contribute to AN-specific pathologies such as poor weight gain and neuropsychiatric abnormalities. To address this, we employed a murine model consisting of germ-free (GF) mice that had been colonized with the microbiota derived from two distinct cohorts: patients with restricting-type AN (gAN) and healthy controls (gHC). As a result, we observed that gAN mice exhibited a discernible reduction in body weight gain and an elevated manifestation of anxiety-related behaviors, as assessed through measures such as marble-burying and open field tests, when compared with gHC mice. These findings suggest that the persistent gut dysbiosis encountered in individuals with AN may play a contributory role in the manifestation of both inadequate weight gain and behavioral abnormalities within this population. A more recent study conducted by Fan et al. [35] demonstrated that GF mice transplanted with stools from individuals with AN and subjected to an energy-restricted diet exhibited a progressively diminished rate of weight gain, compared with mice transplanted with stools from healthy counterparts. Additionally, these AN-transplanted mice displayed heightened expressions of genes associated with appetite suppression in the hypothalamus and an upregulation of genes linked to thermogenesis in adipose tissue. However, the precise causal links between dysbiosis and the specific pathologies characteristic of AN remain to be fully elucidated. Additional investigations are requisite to clarify these associations, particularly through subsequent human studies.

Next-generation sequencing (NGS) provides an overview of the dominant microbial populations in the intestinal ecosystem. Although this technique is accurate when the sequencing has sufficient depth, it has inherent limitations in the accuracy of quantification of low-abundance bacterial groups. By targeting rRNA molecules, the YIF-SCAN^®^ has 100–1,000 times the sensitivity of conventional PCR methods, enabling microbiota analysis with a strikingly wide dynamic range [36]. Moreover, the YIF-SCAN^®^ method offers several advantages, including its capacity for rapid and straightforward operation, making it suitable for analyzing multiple samples efficiently [27, 29]. In fact, a recent study emphasized the need for a quantitative perspective to accurately characterize host–microbe interactions [37]. Therefore, the current results provide valuable quantitative information on how and to what extent gut microbes respond to weight gain.

In our previous study, the detection rate of *Lactiplantibacillus* spp. was significantly lower in individuals with AN than in control women [18]; however, the detection rate in the AN group significantly increased after 2 or 3 months of inpatient treatment. Recently, Schwarzer et al. [38, 39] investigated the effects of *Lactobacillus plantarum* strain WJL (LpWJL) supplementation on growth impairment induced by undernutrition in mice. LpWJL supplementation in malnourished mice reversed the stunted postnatal growth and weight loss. In addition, this improvement in growth parameters coincided with increased circulating levels of insulin and insulin-like growth factor 1. These findings provide valuable insights into the potential role of *Lactiplantibacillus* spp. in mitigating growth impairment associated with AN. Nonetheless, this study used a mouse model; therefore, the results reported by Schwarzer et al. are difficult to apply to the human condition of undernourished adults, such as individuals with AN. Further research is necessary to determine the relevance and efficacy of *Lactiplantibacillus* spp. supplementation in human AN.

In this study, an increase in the number of *Bifidobacterium* during inpatient treatment in individuals with AN was associated with favorable weight gain outcomes one year after the start of inpatient treatment. These findings suggest that *Bifidobacterium* may play a beneficial role in promoting weight restoration in this population. One possible explanation for the observed relationship between the number of *Bifidobacterium* and weight gain could be the involvement of *Bifidobacterium* in gut barrier function and inflammation regulation. Dysbiosis of the gut microbiota in AN may compromise gut barrier integrity, leading to increased gut permeability and systemic inflammation [40–42]. *Bifidobacterium*, known for their potential to strengthen the gut barrier and reduce inflammation [43, 44], could potentially contribute to improved nutrient absorption and reduced systemic inflammation, thereby facilitating weight gain in individuals with AN. However, the mechanisms underlying this association remain unclear and require further investigation.

If certain bacteria have positive or negative effects on renourishment in individuals with AN, targeted strategies to augment or diminish their abundances prior to or during clinical renourishment could potentially result in more effective interventions for individuals with AN [45]. One plausible method to achieve this is through the application of precision nutrition, as suggested by Zeisel [46]. Therefore, the utilization of microbiota-directed complementary foods may be a more effective therapeutic option in individuals with AN, as demonstrated in children with moderate acute malnutrition [47]. Alternatively, as demonstrated in a recently published case report [48], fecal microbiota transplantation may be useful for weight gain in patients with recurrent AN.

This study has several limitations. First, the sample size was small owing to our inclusion criteria which were strictly set to exclude individuals with complications of physical diseases or histories of taking antibiotics or psychotropic medications. However, this may limit the generalizability of our findings and undermine the validity of this study. Second, the YIF-SCAN® only covers selected bacteria that can be detected with a specific primer; however, the conventional bacterial 16S rDNA sequencing method represents the gold standard within this field and offers a more comprehensive analysis than the YIF-SCAN® approach. Further investigations are necessary employing both the bacterial 16S rDNA sequencing method together with the YIF-SCAN®. Additionally, because this was an observational study, causality could not be established. Further studies, including randomized controlled trials, are required to confirm the potential therapeutic effects of *Bifidobacteriu*m or *Lactiplantibacillus* spp. supplementation in patients with AN.

In conclusion, our study offers significant insights into the correlation between AN and the gut microbiota by demonstrating that individuals with AN may continue to exhibit persistent intestinal dysbiosis despite weight restoration. Further understanding of the complex interplay between AN and the gut microbiota may pave the way for the development of novel nutritional approaches to improve treatment outcomes in individuals with AN.

## Supporting information

S1 Table Sequences of the primers and detection limits of each bacterium. (DOCX)

S2 Table Comparison of bacterial counts between individuals with ANR and ANBP at baseline. (DOCX)

S3 Table Comparison of bacterial counts between the control subjects (CON) and individuals with anorexia nervosa (AN) at baseline. (DOCX)
