# Supplementary material for: Persistence of gut dysbiosis in individuals with anorexia nervosa
Source: PLoS One. 2023 Dec 20;18(12):e0296037. doi: 10.1371/journal.pone.0296037 (PMC10732397; doi:10.1371/journal.pone.0296037)
Supplement: S1 Table — (DOCX) [file pone.0296037.s001.docx]

**Supplementary Table 1. Sequences of the primers and detection limits of each bacterium**

| **Target bacteria** | **Primer** | **Sequence(5’-3’)** | **Detection limits**  **(Log10/g feces)** | **Reference** |
| --- | --- | --- | --- | --- |
| *Blautia (Clostridium) coccoides* group | g-Ccoc-F  g-Ccoc-R | AAATGACGGTACCTGACTAA  CTTTGAGTTTCATTCTTGCGAA | 5.0 | Matsuki et al., 2002 |
| *Clostridium leptum* subgroup | sg-Clept-F  sg-Clept-R3 | GCACAAGCAGTGGAGT  CTTCCTCCGTTTTGTCAA | 5.0 | Matsuki et al., 2004 |
| *Bacteroides fragilis* group | g-Bfra-F  g-Bfra-R | ATAGCCTTTCGAAAGRAAGAT  CCAGTATCAACTGCAATTTTA | 5.0 | Matsuki et al., 2002 |
| *Bifidobacterium* | g-Bifid-F  g-Bifid-R | CTCCTGGAAACGGGTGG  GGTGTTCTTCCCGATATCTACA | 5.0 | Matsuki, et al. 1998 |
| *Atopobium* cluster | c-Atopo-F  c-Atopo-R | GGGTTGAGAGACCGACC  CGGRGCTTCTTCTGCAGG | 5.9 | Matsuki et al., 2004 |
| *Prevotella* | g-Prevo-F  g-Prevo-R | CACRGTAAACGATGGATGCC  GGTCGGGTTGCAGACC | 5.1 | Matsuki et al., 2002 |
| *Clostridium perfringens* | s-Clper-F  ClPER-R | GGGGGTTTCAACACCTCC  GCAAGGGATGTCAAGTGT | 2.3 | Matsuda et al., 2009; Kikuchi et al., 2002 |
| *Clostridioides (Clostridium) difficile* | Cd-lsu-F  Cd-lsu-R | GGGAGCTTCCCA TAC GGG TTG  TTG ACT GCC TCA ATGCTT GGG C | 2.4 | Matsuda et al., 2012 |
| Enterobacteriaceae (formerly taxonomic nomenclature) | En-lsu3F  En-lsu3’R | TGCCGTAACTTCGGGAGAAGGCA  TCAAGGCTCAATGTTCAGTGTC | 3.9 | Matsuda et al., 2007 |
| *Staphylococcus* | g-Staph-F  g-Staph-R | TTTGGGCTACACACGTGCTACAATGGACAA  AACAACTTTATGGGATTTGCWTGA | 3.0 | Matsuda et al., 2009 |
| *Enterococcus* | Ec-ssu1 F  Ec-ssu1R | GGATAACACTTGGAAACAGG  TCCTTGTTCTTCTCTAACAA | 3.0 | Matsuda et al., 2009 |
| *Streptococcus* | F  R | GCTTAGAAGCAGCTATTCATTC  GGATACACCTTTCGGTCTCTC | 3.0 | Sakaguchi et al., 2010 |
| *Lactobacillus* (*Lactobacillus gasseri* subgroup) | sg-Lgas-F  sg-Lgas-R | GATGCATAGCCGAGTTGAGAGACTGAT  TAAAGGCCAGTTACTACCTCTATCC | 2.7 | Matsuda et al., 2009 |
| *Lactiplantibacillus* (*Lactobacillus plantarum* subgroup) | sg-Lpla-F  sg-Lpla-R | CTCTGGTATTGATTGGTGCTTGCAT  GTTCGCCACTCACTCAAATGTAAA | 2.6 | Matsuda et al., 2009 |
| *Limosilactobacillus except L. fermentum (Lactobacillus reuteri* subgroup) | sg-Lreu-F  sg-Lreu-R | GAACGCAYTGGCCCAA  TCCATTGTGGCCGATCAGT | 3.0 | Matsuda et al., 2009 |
| *Lacticaseibacillus* (*Lactobacillus casei* subgroup) | sg-Lcas-F  sg-Lcas-R | ACCGCATGGTTCTTGGC  CCGACAACAGTTACTCTGCC | 3.5 | Matsuda et al., 2009 |
| *Liquorilactobacillus and Ligilactobacillus* (*Lactobacillus ruminis* subgroup) | sg-Lrum-F  sg-Lrum-R | CACCGAATGCTTGCAYTCACC  GCCGCGGGTCCATCCAAAA | 2.3 | Matsuda et al., 2009 |
| *Latilactobacillus* (*Lactobacillus sakei* subgroup) | sg-Lsak-F  sg-Lsak-R | CATAAAACCTAMCACCGCATGG  TCAGTTACTATCAGATACRTTCTTCTC | 2.9 | Matsuda et al., 2009 |
| *Limosilactobacillus* (*Lactobacillus fermentum*) | LFer-1  LFer-2 | CCTGATTGATTTTGGTCGCCAAC  ACGTATGAACAGTTACTCTCATACGT | 4.0 | Matsuda et al., 2009 |
| *Levilactobacillus* (*Lactobacillus brevis*) | s-Lbre-F  s-Lbre-R | ATTTTGTTTGAAAGGTGGCTTCGG  ACCCTTGAACAGTTACTCTCAAAGG | 2.6 | Matsuda et al., 2009 |

Kikuchi, E., Miyamoto, Y., Narushima, S. and Itoh, K. (2002). Design of species-specific primers to identify 13 species of *Clostridium* harbored in human intestinal tracts. Microbiol. and Immunol. 46, 353-8. doi: 10.1111/j.1348-0421.2002.tb02706.x

Matsuda, K., Tsuji, H., Asahara, T., Kado, Y., and Nomoto, K. (2007). Sensitive quantitative detection of commensal bacteria by rRNA-targeted reverse transcription-PCR. Appl. Environ. Microbiol. 73, 32-9. doi:10.1128/AEM.01224-06.

Matsuda, K., Tsuji, H., Asahara, T., Matsumoto, K., Takada, T., and Nomoto, K. (2009). Establishment of an analytical system for the human fecal microbiota, based on reverse transcription-quantitative PCR targeting of multicopy rRNA molecules. Appl. Environ. Microbiol. 75, 1961-9. doi:10.1128/AEM.01843-08.

Matsuda, K., Tsuji, H., Asahara, T., Takahashi, T., Kubota, H., Nagata, S., et al. (2012). Sensitive quantification of *Clostridium difficile* cells by reverse transcription-quantitative PCR targeting rRNA molecules. Appl. Environ. Microbiol. 78, 5111-8. doi:10.1128/AEM.07990-11.

Matsuki, T., Watanabe, K., Tanaka, R., and Oyaizu, H. (1998). Rapid identification of human intestinal bifidobacteria by 16S rRNA-targeted species- and group-specific primers. FEMS Microbiol. Lett. 167, 113-21. doi: 10.1111/j.1574-6968.1998.tb13216.x.

Matsuki, T., Watanabe, K., Fujimoto, J., Miyamoto, Y., Takada, T., Matsumoto, K., et al. (2002). Development of 16S rRNA-gene-targeted group-specific primers for the detection and identification of predominant bacteria in human feces. Appl. Environ. Microbiol. 68, 5445-51. doi:10.1128/AEM.68.11.5445.

Matsuki, T., Watanabe, K., Fujimoto, J., Takada, T., and Tanaka, R. (2004). Use of 16S rRNA gene-targeted group-specific primers for real-time PCR analysis of predominant bacteria in human feces. Appl. Environ. Microbiol. 70, 7220-8. doi:10.1128/AEM.70.12.7220-7228.2004.

Sakaguchi, S., Saito, M., Tsuji, H., Asahara, T., Takata, O., Fujimura, J., et al. (2010). Bacterial rRNA-targeted reverse transcription-PCR used to identify pathogens responsible for fever with neutropenia. J. Clin. Microbiol. 48, 1624-8. doi:10.1128/JCM.01724-09.
