# Supplementary material for: Persistence of gut dysbiosis in individuals with anorexia nervosa
Source: PLoS One. 2023 Dec 20;18(12):e0296037. doi: 10.1371/journal.pone.0296037 (PMC10732397; doi:10.1371/journal.pone.0296037)
Supplement: S2 Table — (DOCX) [file pone.0296037.s002.docx]

**Supplementary Table 2. Comparison of bacterial counts between individuals with ANR and ANBP at baseline†**

|  | Log_10_ cells/g feces | | p value |
| --- | --- | --- | --- |
|  | ANBP (n = 6) | ANR (n = 7) |  |
| Total bacteria | 10.8 ± 0.2 | 10.5 ± 0.5 | 0.3498 |
| *Blautia coccoides* group | 9.5 ± 0.5 | 9.3 ± 0.4 | 0.5157 |
| *C. leptum* subgroup | 9.7 ± 0.5 | 9.6 ± 0.6 | 0.7202 |
| *B. fragilis* group | 9.6 ± 0.5 | 9.7 ± 0.5 | 0.8062 |
| *Bifidobacterium* | 10.6 ± 0.5 | 9.6 ± 1.6 | 0.1087 |
| *Atopobium* cluster | 8.7 ± 1.8 | 9.3 ± 0.7 | 0.6674 |
| *Prevotella* | 5.7 ± 0.4 | 6.6 ± 0.7 | 0.0662 |
| *Enterobacteriaceae* | 7.5 ± 0.8 | 7.1 ± 1.0 | 0.5650 |
| *Enterococcus* | 7.3 ± 1.3 | 6.5 ± 0.9 | 0.2840 |
| *Staphylococcus* | 5.7 ± 0.7 | 5.6 ± 1.1 | 0.8859 |
| *Streptococcus* | 8.0 ± 0.8 | 8.6 ± 0.4 | 0.1269 |
| *Clostridioides difficile* | 5.8 ± 0.7 | ND | NT |
| *C. perfringens* | 4.8 ± 3.3 | 5.2 ± 1.0 | NT |
| Total lactobacilli | 7.1 ± 2.1 | 5.1 ± 2.2 | 0.1699 |
| *Lactobacillus* | 5.8 ± 2.2 | 4.2 ± 1.0 | 0.3913 |
| *Limosilactobacillus except L. fermentum* | 5.2 ± 1.9 | 3.9 | NT |
| *Liquorilactobacillus and Ligilactobacillus* | 5.6 ± 1.3 | 7.5 | NT |
| *Lactiplantibacillus* | 4.0 ± 1.6 | 2.7 | NT |
| *Latilactobacillus* | 3.9 ± 0.5 | 3.7 ± 1.0 | NT |
| *Lacticaseibacillus* | 7.2 ± 2.0 | 5.9 ± 1.9 | NT |
| *Levilactobacillus* | 3.9 | ND | NT |
| *Limosilactobacillus* | 8.7 | ND | NT |

**†**All data are expressed as means ± SDs. ND, not detected; *C, Clostridium; B, Bacteroides*. NT means that statistical analyses are "not tested" because the number of samples in each group is smaller than 4. The total count of lactobacilli (Total lactobacilli) is expressed as the sum of the counts of *Lactobacillus, Lactiplantibacillus, Limosilactobacillus except L. fermentum, Lacticaseibacillus, Liquorilactobacillus and Ligilactobacillus, Latilactobacillus, Limosilactobacillus,* and *Levilactobacillus*. The results were corrected using the Bonferroni test, based on the number of trials; therefore, p values of <0.0038 (0.05/13) were considered significant.
