# Supplementary material for: Persistence of gut dysbiosis in individuals with anorexia nervosa
Source: PLoS One. 2023 Dec 20;18(12):e0296037. doi: 10.1371/journal.pone.0296037 (PMC10732397; doi:10.1371/journal.pone.0296037)
Supplement: S3 Table — (DOCX) [file pone.0296037.s003.docx]

**Supplementary Table 3. Comparison of bacterial counts between the control subjects (CON) and individuals with anorexia nervosa (AN) at baseline†**

|  | Log_10_ cells/g feces | | p value |
| --- | --- | --- | --- |
|  | CON (n = 21) | AN (n = 13) |  |
| Total bacteria | 11.1 ± 0.5 | **10.5 ± 0.5*** | **0.0026** |
| *Blautia coccoides* group | 10.0 ± 0.4 | **9.4 ± 0.4*** | **0.0016** |
| *C. leptum* subgroup | 10.4 ± 0.7 | **9.7 ± 0.5*** | **0.0018** |
| *B. fragilis* group | 10.5 ± 0.6 | **9.6 ± 0.5*** | **0.0002** |
| *Bifidobacterium* | 10.3 ± 0.7 | 10.1 ± 1.2 | 0.7789 |
| *Atopobium* cluster | 9.3 ± 0.8 | 9.1 ± 1.3 | 0.8041 |
| *Prevotella* | 6.9 ± 1.3 | 6.2 ± 0.7 | 0.2268 |
| *Enterobacteriaceae* | 7.1 ± 0.9 | 7.3 ± 0.9 | 0.6728 |
| *Enterococcus* | 6.2 ± 1.2 | 6.9 ± 1.2 | 0.1309 |
| *Staphylococcus* | 5.3 ± 0.9 | 5.7 ± 0.9 | 0.3210 |
| *Streptococcus* | 9.0 ± 0.7 | 8.3 ± 0.7 | 0.0062 |
| *Clostridioides difficile* | ND | 5.8 ± 0.7 | NT |
| *C. perfringens* | 4.9 ± 1.3 | 5.1 ± 1.6 | 0.6931 |
| Total lactobacilli | 6.0 ± 1.1 | 6.2 ± 2.3 | 0.7210 |
| *Lactobacillus* | 5.4 ± 1.2 | 5.1 ± 1.9 | 0.2811 |
| *Limosilactobacillus except L. fermentum* | 4.9 ± 1.0 | 4.9 ± 1.7 | 0.9417 |
| *Liquorilactobacillus and Ligilactobacillus* | 4.2 ± 1.1 | 6.1 ± 1.4 | 0.0726 |
| *Lactiplantibacillus* | 4.0 ± 0.8 | 3.5 ± 1.3 | 0.5536 |
| *Latilactobacillus* | 4.4 ± 1.3 | 3.8 ± 0.7 | 0.4137 |
| *Lacticaseibacillus* | 5.8 ± 1.4 | 6.7 ± 1.9 | 0.3566 |
| *Levilactobacillus* | 5.3 ± 0.3 | 3.9 | NT |
| *Limosilactobacillus* | 4.6 ± 0.7 | 8.7 | NT |

**†**All data are expressed as means ± SDs. The CON group is identical to that of healthy women reported in our previous literature (Morita et al. *PlosOne* *10*(12), e0145274). Total lactobacilli is expressed as the sum of the counts of *Lactobacillus, Lactiplantibacillus, Limosilactobacillus except L. fermentum, Lacticaseibacillus, Liquorilactobacillus and Ligilactobacillus, Latilactobacillus, Limosilactobacillus,* and *Levilactobacillus*. An asterisk (* p < 0.0026 [0.05/19]) indicates a significant difference between the AN and CON groups after the Wilcoxon-Mann-Whitney test, followed by the Bonferroni test, based on the number of trials. ND, not detected; *C, Clostridium; B, Bacteroides*. NT means that statistical analyses were "not tested" because the number of samples in each group is <4.
